# Supplementary material for: Patterns of Intron Gain and Loss in Fungi
Source: PLoS Biol. 2004 Nov 30;2(12):e422. doi: 10.1371/journal.pbio.0020422 (PMC532390; doi:10.1371/journal.pbio.0020422)
Supplement: Table S1 — Also available at http://genes.mit.edu/NielsenEtAl/. (4.3 MB ZIP). [file pbio.0020422.st001.zip › NielsenEtAl/html/973.html]

AN6551.1.NCU03811.1.MG04037.1.FG06097.1


```
 CLUSTAL W (1.82) Multiple Sequence Alignments - Introns Inserted


Sequence 1: MG04037.1	1508 aa
Sequence 2: FG06097.1	1911 aa
Sequence 3: NCU03811.1	1840 aa
Sequence 4: AN6551.1	1734 aa
Alignment Length: 1995 aa
Number Identitical Residues: 436 aa
Alignment Score (without introns) 30432


MG04037.1 	-------------------------------------------~----------------
NCU03811.1	----MDYSQLRAAALQNGEDEEAVTVDTRALIDKVLARYSGEW~TTLRELIQNAADAQAT
FG06097.1 	----MDYSKLRAAALRDGEDEEAVTVDTRALIDKVLARYSGEW~TTLRELIQNAADAQAK
AN6551.1  	MTQNVDFSALKARTMRSGEDEEAVTVDTRGLISKVLARYSGQW2TVLREMIQNAADANAT
          	 :.. . :   : :  ......: : .: .  ..  :  :..  :   .  ..::.:.:.

MG04037.1 	--~---------------------------------------------------------
NCU03811.1	TV~KIKWETIPSVSVPAPQTTDPSELLKHTLTHHTLRRLLVQNDGQPFANTDWARLKRIA
FG06097.1 	TV~SVKWETLPSTQVPLPSTTNRSEILKHTLSNHTLRRLVVQNDGQPFTKTDWGRLKRIA
AN6551.1  	KV1TIKFETLPSKTVPFPSTTDRTSLIKHTISHHTIKRLLISNNGLLFNEKDWARLKRIA
          	.  . . .: .:   . ..::. :.  . : :  :      ....    ... .  .  :

MG04037.1 	-----------~------------------------------------------------
NCU03811.1	EGNPDETKIGA~FGVGFYSVFADCEEPFVSSGKEAMAFYWKGNALFTRKLQLPVEQHSRE
FG06097.1 	EGNPDETKIGA~FGVGFYSVFADCEEPFVSSGDQAMAFYWKGNALFTKKSQIPADQATHH
AN6551.1  	DGNPDETKIGA2FGVGFYSVFEDCEEPFVSSGSDAMAFYWKENALFTRRLQLN-EQANSE
          	......:. .:  . .  :   .....  ::...: :   . .:  :   .   .. .  

MG04037.1 	------------------------------------------------------------
NCU03811.1	TAFVLDYRNTTTPLPNLLSVSQFLGTSLTFVSLQKIEFWIDDWRVLALQKKSSPSAQIPI
FG06097.1 	TTFVLDYRNNTTPLPNLLSVSQFLATSLTFVALEHIEFWIDDYRVLSLKKKSSPSVELPL
AN6551.1  	TTFVLDYRNDTSPIPSLMQLCQFLSSSLTFVNLECIELWLDDWNILRLAKKAAPGIALAL
          	::   .  . ::. ..  . ..  .:: :    .  .   ..       ..::..   . 

MG04037.1 	-----------------------------MSVLGWKPKAASAAKSNDTYGFNSSDMPSLR
NCU03811.1	PRDLETRTKDGLMKLTAAEQTSTQIDASFMSVLGWKPKPVSSSNKSTESAY-TPEPSGLK
FG06097.1 	PRDLEARTKDGLMKVTSVDRTSTQIDASYMAAIGWKPQAASSSTKTDSYG--APEAPSLR
AN6551.1  	PKDIETKTQEGLMKITSVTREVAQVDAAWMQVVEWNPNSSTLVEG------IRDTTSSLR
          	. . .: :...  . ::     :. .:: * .: *:*:. :               ..*:

MG04037.1 	SFFSRLTSSASQSGLRGKQAKEEKQTSEPVVEDLTITSTSSVFLRVTSAVITTSVPTSFA
NCU03811.1	GFFSRFTSSAAQTAQRTKAAKEEKEIQEAIAEDLTAVSTSTIFLRITAAQIATSVSSSFA
FG06097.1 	SFFARLTSTASQAGIRTKAQTEENAAQVAIAEDVTKLSTSTIFLRATSASIRTSVATNFA
AN6551.1  	SFLSRLTQGSS-----SKVADTQKKEAADDTGDLTKISTATIFLHINTGSIQASISQSLG
          	.*::*:*. ::      *    ::      . *:*  **:::**: .:. * :*:. .:.

MG04037.1 	AELERATKKPPPRTTKLSILTSSYDEAQASKNSNDD--SKNTVDVFTSVLPSKKPGGKIF
NCU03811.1	AELERATKKPPPKTTRIALLTSSYDETVASEESVASNKAAKAIDIFSSVLPSKKPGGRVF
FG06097.1 	SELERATKKPPPKTTKIAILTSSYDEAQASQEAASPGALGKATDMFASVLPNKKPGGRIF
AN6551.1  	SELERATRKPPPKKTSIAVLTPSYDTSLAS--------SSSQAEFLSTILPSK--GGRVF
          	:******:****:.* :::**.*** : **          .  :.::::**.*  **::*

MG04037.1 	IGFPTTQTTGGGMHISAPSVIPTVEREAIDLNARWVRSWNIEMLRVAGIMTRLAFSTEMA
NCU03811.1	IGFPTTQTTGAGMHISAPSVIPTVEREAIDLNARWVRTWNIEMLRVAGIISRLAFVNDMS
FG06097.1 	IGFPTMQTTGAGMHVSAPSIIPTVEREAIDLNARWVRTWNLEILRAAGIITRLAFANEMS
AN6551.1  	IGFPTQQTTGLNAHISAPSVIPTVERESIDLNSRYIRKWNTEMLRAAGIICRIAWSAEMA
          	***** **** . *:****:*******:****:*::*.** *:**.***: *:*:  :*:

MG04037.1 	DLQTKVQRAVNG------AQITTADISKFIPEALHILETYTFRDSTPSQYVAQIIKESFW
NCU03811.1	DLNEKLKRLSNSKNNKKESGYALEDVQKFVPEALHILKTYSFEDSTPSAQVAKIIEEAFW
FG06097.1 	DLSSRIRQVMAKE-----PKFSPAVIAKYMPEALHILKTFTFGDSTPSGQVGQIIEEAFW
AN6551.1  	SVKNRIISGKDPS---KQSKIRKADITTVLPEAIHTANQFVFRESTPLSVLGQIIEDAFW
          	.:. ::      .   ...      : . :***:*  : : * :***   :.:**:::**

MG04037.1 	TAFKKAYIEVFSSRGVLLSTKVRMGSDELAKFVDGIPIIIKEMEEAP--FVRELREYGLI
NCU03811.1	TSYRKASIEVFSCQGVLQTDKVRLFSEDYAKFLSGIPIILKDMQAAP--FIQKLIDYGLL
FG06097.1 	MCFKKASIETYSTRGVLQTADVRIASDEMSKFVDSIPVVPEEMKGVP--FVKKLIDFGLI
AN6551.1  	TCNKNASIEVISTCGVVHNHQARIATKDLT-FLDSIPVLPDEFVEGSKEFVKKLTLLGLV
          	 . ::* **. *  **: . ..*: :.: : *:..**:: .::   ...*:::*   **:

MG04037.1 	DEITVTDIRKELEAKALNKDQLVHFLTWAGKRAVTGELDPPSRQTLLDVAIATISEGGDQ
NCU03811.1	EHITVDDVVKELGSKAMDKHQLHHFVVWLAKTAASGELKYHHIQHVLDVAVATVSDDGNG
FG06097.1 	SHITVTDVKKELETKAMNKVQLMNFISWAGKKSLSGELDPGSRSALLEVAVGTMSDDGEQ
AN6551.1  	TEVTVTDIKRELETCPLRSSQITEFLSWLARRTVSGQLDSYSARSILNVAVASADENDTD
          	 .:** *: :** : .: . *: .*: * .: : :*:*.      :*:**:.: .:..  

MG04037.1 	GEVIALTTIRNYLSANKIPASLPLPPTTLPYALTCNIPAAHLEMLGWEKLEIVPWLRYLL
NCU03811.1	GEIIALGSIKNYLN-NRIPATLPIPPTTIPVAFTADIPEVQLQSIGWEPLGIVPWLRFLI
FG06097.1 	GEIIALGSITNHLITNRIPASLPIPPTTIPHALTANSQPAELRALGWEPLEVVPWLRFLF
AN6551.1  	TGLIVFSGVSLFLNPQRIPADLPLPPAVMPFKYTKSLSKKDLESFGWEELQIVPWLCWLV
          	  :*.:  :  .* .::*** **:**:.:*   * .    .*. :*** * :**** :*.

MG04037.1 	ESAGSK---DDEQNITKSAKFAAQILVVLSKNWENLSQPSRNSVVSLLQNKTVVPTKMGM
NCU03811.1	ASGSSN---GEEHNLTKSSKFSVQVLSVLSKNWDNLSPSDKSSVVSALEHLPVIPTKLGM
FG06097.1 	DTNSSR---SEEQNLTRSPKFAIQVLTVLSKNFENSSPGARITIVSLLQANTVVPTKQGM
AN6551.1  	SNAGNRDVLPQTQDITKCPSFAAQVLPVISKQWETLGQSSKQDVIDQLQAHTVIPTKIGM
          	 . ....   : :::*:...*: *:* *:**:::. .   :  ::. *:  .*:*** **

MG04037.1 	KKPSESFFPTVKLFDDLPIVQGCQGVKDKFLVAIGVRKTLDLETIFTRLLTPEKEGS---
NCU03811.1	RKPKESFFSSVKLFHDLPTIEGCDRLKEKFLVALGVRKTLDLETIFTRLLNPAPAAEGE-
FG06097.1 	KRPTESFFPIVKLFDDLPIIQGCDKIKEKFLVTIGVRKTVDLETIFTRLLNASSEGQ---
AN6551.1  	KCPTEAYFSSVRLFDDLPVVHGLQGVKEKLLTALGVRKTVELGVIFERLLNAPGSSDGDK
          	: *.*::*. *:**.*** :.* : :*:*:*.::*****::* .** ***..   .....

MG04037.1 	---SKWSHMELIKYLASVRDDIPPEDLRRLKESKICPAEAGP--AGMEPTKGTHQLFKVS
NCU03811.1	AEAKKWSHVELIKYLASVKNDIPAEDMKRLKQSKLCPAEDRTFFGGPESRKGTEKLYKLS
FG06097.1 	---QKWSHVELIKYLASVREDIPGEDLSKLKQTRFCPAEAGP--KGMESTKATETLYKVS
AN6551.1  	SSQGKWSHVDLIRYLASVSSDIPASDIKRLKDTNFCTAEPIIDHDGSR--RPNEDRYKVQ
          	:.  ****::**:***** .*** .*: :**::.:*.**      * .  : ..  :*:.

MG04037.1 	ELFEPKDTLRTLKLPILQWPGPPGSFRPSSPEGRFLFSLGLRPHPSVPELVEMMASDDPS
NCU03811.1	ELYEPKDSLRLLQLPTLHWPG---IWKANSPEGAFLFSLGLRPYPAVPELIDMMASPDAD
FG06097.1 	ELFEPKDAIRGLRLPVLQWPGPPGSYRPGSHEARFLNSLGLRPYPSVPELVELMAGKDEA
AN6551.1  	QLYEPNDALRALRLPILEWPA---KFTSSSPEGRFLARLGLRTFPQSTVLTRIMAAAAEH
          	:*:**:*::* *:** *.**.    : ..* *. **  ****..*  . *  :**.    

MG04037.1 	----MRASSMTYFIANHHINGYASFNIGATKRAILPIQGN-------EKALVSPSECYAD
NCU03811.1	----TRKHSMAYFINNYQLNQYGTYNLSRIDNAILPLQGN-------EKKLVRPSACFTN
FG06097.1 	----LRVSSMTYFLANHQINGYGSFQLGGSPKAIMPLQG--------STKLVPPSQCFYN
AN6551.1  	NDWALHGKAMSYYVTEFENNGYGAIDCGSINDEFLPVEQINDSGAEKRYKVSAPSKCFTD
          	.. : :  :*:*:: :.. * *.: : .     ::*::  ..:.:..   :  ** *: :

MG04037.1 	EGGAVLGFNIIAKNLQVHASKFGVTRDPPITVCVSKLVTKPPHDRQSAMTLFRYFATRLG
NCU03811.1	PDAAILGYDILRRDLHDHASKFGVARDPSILECVGRLIASPPKDHQEAVRVFGYFTTRIA
FG06097.1 	PRASILGFNILRRDLHDHAVKFGVARDPPMADCVNRLLASPPQDHQNAVALFSYFASRIA
AN6551.1  	EGAALFGYDILRKDLHRHASKLGVQRHPKLSNCLDTLIRLPPSTVREARSLYRYLAGRVS
          	  .:::*::*: ::*: ** *:** *.* :  *:. *:  **   :.*  :: *:: *:.

MG04037.1 	DLGGNHLAKLRDSNIVPVTRTERGS-LKGSSIVVMLKPQNCYLGGSSTYSDIFDFVDFGS
NCU03811.1	ELGLSHVTKLRSSAIIPVTRRKVSSDEKGDTKVVYITPPNCYLGTSSTYGDIFDFVDFGP
FG06097.1 	DLGENYLFRLRDAPIVPITRTSQST-EKPKRTQVHVSPSRVYLGSSTTYGDIFDFVDFGP
AN6551.1  	ELTPRDIDRVGRAEIVPVFAENNKG-----STMHRVAPSLCYLGEGEDYKDIFDFVDFGQ
          	:*    : ::  : *:*:   .             : *   *** .  * ********* 

MG04037.1 	DANAFLFKCGAKSEPTKPEIAQMACSEPARLLSILQSPEKYLNLLRTLAEDMATIKRDRE
NCU03811.1	NANVFLFKCGAKEEPTKPEIALMACSEPARLLSTLQSSEKYLNLLRSLADEIATLKRDKE
FG06097.1 	EANAFLFQCGAKSEPTKLEVAHMACTEPARLLGVLQSSEKYLDLLKSLAEASSTLHRDKD
AN6551.1  	EANLFLMAVGSKREPTKIELAQMIVREPARISSTFQSGEKYLKLLRTLAEDMSLLRKNKE
          	:** **:  *:* **** *:* *   ****: . :** ****.**::**:  : ::::::

MG04037.1 	LWAKMKVSAFLLASKEIASTK----------SSKDIDADEDEAPIKQYQLAAPGQIVVVD
NCU03811.1	LFKKMTSSAWLLGSRDISNTK----------EEPD--SGDYDAPIKQYQLASPQKITILD
FG06097.1 	LWRRMKSAPFLLAYKELAPPK----------KQSD-DLEEEDEPIRQYQLASASQIVILD
AN6551.1  	LWQEMKRSAFLLASKDITSTAQVGDGAKKIVDNSDDEDEAEDGGIREWALTMAKETIVVD
          	*: .*. :.:**. :::: . . ...:..  .. * .    :  *::: *: . :  ::*

MG04037.1 	DYISYRLFKDSLMC--~-APEEDALESFYL~ALGSQMLGNLVQEDLKVGPHSEKQEGAVW
NCU03811.1	DIICYRLFKDSLLF--~-APEDDALEAFYA~ALGSEAISSKVSMEHRIGPAAEKQDAAVT
FG06097.1 	DIISYRLFKDQLIC--~-APEEDALETFYL~SLGAQRLSSIVREEIRIGPHSDRQKLALS
AN6551.1  	DYQSFILFKDHVLAGK2PAPQEEILENFYL1ALGALPLSQLVEERANWGAPATDQRLAVK
          	*  .: **** :: .. .**::: ** **  :**:  :.. *    . *. :  *  *: 

MG04037.1 	LRKHVLERSKLFLYEYTKTNNHAIKHDVKWLEKNLMVEVVRNVALRRSLRGQTQSHMEKR
NCU03811.1	MRKLVIERSKIFLFEYSKYKKSSIKHDARWLEKNLQVKAVTSLALRRSLKGYNQQHTEKR
FG06097.1 	LRKHVVERSKIFLFEYANYRRDAIKHDAKWLEKNLTVDVVHSVALRRTLQGQYQTHTEKR
AN6551.1  	LLKLIKERSRLFLHDQP---ADSIRHDSRWLDKNLNVQVVQSITLTRSLRNRGISYRQER
          	: * : ***::**.: .     :*:** :**:*** *..* .::* *:*:.    : ::*

MG04037.1 	SAASVQTRNGWVLYVAAEGRPDMYQVGQAVCHLLLTRPNQQAYFFFEPFLKLDLLDLRAR
NCU03811.1	SAAAVYRDGNWILYISTHSRPDLYQVGQAICTMLLSRHGQQAYFFIEPFLKLDLLELRAR
FG06097.1 	SAASKKTNNGWIMYVADEAKPDMYQIGQAICQMLLSRPNQQAYLFFEPFLTLDLYGLRNR
AN6551.1  	KAIVTEVSRECIMRICP-EKYDFYEISQALSSLILARPKLHSALTLEMLLKTDLLELRGR
          	.*         :: :.   : *:*::.**:. ::*:*   :: : :* :*. **  ** *

MG04037.1 	GY~NVDRILRAKAAEARIAEEERRKALEEEQQRIREREQEWRQS--GAAQAEAAARNQRP
NCU03811.1	GY~NVDRILRAKAAEARIAEEERRKALEAEEQRIREREKDWAQQQLATADNAVARAPQTP
FG06097.1 	GY~NVDRILRAKAAEARIAEEERRKALEEEQRQIKEKEQQWGAE--AKAAEAAREATRVP
AN6551.1  	GY0NVDRILKQKAHDAQIAETRRQQQLEEERRRLREKE----------LALAEEARDQIA
          	** ******: ** :*:*** .*:: ** *.::::*:*                   : .

MG04037.1 	KTPE-PQMPGSFAVDSPDSPPHSLPPPQDTRKKGQGLFSNWSKRLGFDRENDE-------
NCU03811.1	RLPDQPRMPGG----FDDSPDHDTAP--QPIKKFGDFFSGIKKSLGLEADEDSNRPPAQQ
FG06097.1 	EPLG-PTMPG-----SWESPEQAKPTQEQQNRKSRGLFSNLTRRLGFDTEQDED------
AN6551.1  	ETQDATSMPGVFP-DSPSSKDVRPNSPTQSPKEKRNLFSTFTKHFSEGNRSSWN------
          	.    . ***  . .  .*      .  :  ::  .:**  .: :.    .. .      

MG04037.1 	-GHKQLQNLIDQPKVPAPQTGSEGGAPGPSQPPKDDGRVTSPAVVQQNLLNAINSTRAHD
NCU03811.1	PGTRAPQQLGPGSGGSGSGSGGGGGGGGGAEGGSNDGRVTSPAVVQQNLLSAIKSTRAHD
FG06097.1 	DSRKELNKFLDKPKEIEAPSG---GSSHSGKRPQDEGRVTSPAVVQQNLLNAVKSTRAYG
AN6551.1  	PFRGETASLPDPPRASPPQLP---TEQVKPPRPEAPVAVNSALQLQKSLHSAIQSSRSHG
          	       .:   .    .               .    *.*.  :*:.* .*::*:*::.

MG04037.1 	SRTLFSPPQTNEVKEQASYCDSTAAHDLRFVADAPQGMRVFVANDLSMDQGKFLEAHSRS
NCU03811.1	ASGVFAPPTQTEITEQSTYCDSTPATNMNFVAEAPGGMRVFLAKDVSVNPGQFLSSNTAG
FG06097.1 	SDSVFSEPQVNEVKEQATYCDKTPAQNITFIAETSNSMKVFVSRDIK-DSSAFLSKHLTN
AN6551.1  	ASGVFSRPQVDRINELKSYCDEKPGHDLEFAATLPCGVNILVARSVE--RSAFLTKNSVG
          	:  :*: *   .:.*  :***.... :: * *  . .:.::::..:.   . **  :  .

MG04037.1 	ITTFEGVLRDIGTIYGLAPTVLHIFYDERGNTIAFNSNGSVFCNLRFFLQLHAERMG---
NCU03811.1	ISKFETLLKDVANVYNLPAHSLHIYYDEQGPTIAFNKSGSIFCNYRFFSQLHLAKLTGGP
FG06097.1 	LNVFSSLLIDVGNVYSMSPKVLHIFYDEAGGTIAFNTGGSIFCNFRFFLQLHAAQIEG--
AN6551.1  	LNLFGSILLDCADVFALRPDTVSIFYDPGSKAIGFNRSGSIFCNYHYFQQLHEKALLQ--
          	:. *  :* * . :: : .  : *:**  . :*.** .**:*** ::* ***   :    

MG04037.1 	---SGNGRTEATTWWWVVIAHELAHNLVQPHNSDHSYYT~-----------ESFIQQFFP
NCU03811.1	DAAGGMGRAEATVWWWVTIAHELAHNLVSPHNSAHSYYT2NQRLTINIKYSESFIQEYFG
FG06097.1 	--RSGQAKAEAATWWWVVLAHELAHNLVSPHNSDHSYYT2-----------ESFIQQYFS
AN6551.1  	--KPTPDRAEAMVYWWVTLSHELAHNLVADHSSAHSYYT2-----------ESFVSQYFT
          	       ::** .:***.::********  *.* *****            ***:.::* 

MG04037.1 	KMMAKAAQWNTS-APAAAAAAAAPSTANAGG---------~------~---NGQ------
NCU03811.1	KMMAMTAMWATQSLSQSQASVGNSLPAPPPPPQQ------~------~------------
FG06097.1 	KIVAKAAQWTANPTPRSQTPSITPPPSGAPPPYSGGLDRN1NTVRTL2TTPNGQKVQGRM
AN6551.1  	RIAAKIAHAATKRPNPEQ----------------------~------~------------
          	:: *  *   :.                                                

MG04037.1 	-------------------------HSLPPPPAYSQQAPARNSLIDL-------------
NCU03811.1	------------------------GTGVLGSIAGNPFHTAHSSLSSVAVR----------
FG06097.1 	IHVTGIVTNQLDSNCSELRQTDLQDHDLTLPITNDFNASHLERISDLSNSHIRIPDGQLP
AN6551.1  	------------------------------------------------------------
          	                                                            

MG04037.1 	------------~-----------------------------------------------
NCU03811.1	------------~-----GQQGQQEPPPPYENGRSLTGGQANGNQGYRSLLD--------
FG06097.1 	NMHRIAFWSLFG1LGARFWQMGIEMRPFFNKSSLWVYPVYAAGGASFGYWLQGVDDRQTD
AN6551.1  	------------~-----------------------------------------------
          	                                                            

MG04037.1 	-------------------------
NCU03811.1	-------------------------
FG06097.1 	TLKERKNILLEKRARKAEKDAQAEA
AN6551.1  	-------------------------
          	
```
